# Human Tissues Exhibit Diverse Composition of Translation Machinery

**DOI:** 10.3390/ijms24098361

**Published:** 2023-05-06

**Authors:** Aleksandra S. Anisimova, Natalia M. Kolyupanova, Nadezhda E. Makarova, Artyom A. Egorov, Ivan V. Kulakovskiy, Sergey E. Dmitriev

**Affiliations:** 1 Belozersky Institute of Physico-Chemical Biology, Lomonosov Moscow State University, 119234 Moscow, Russia; 2 Faculty of Bioengineering and Bioinformatics, Lomonosov Moscow State University, 119234 Moscow, Russia; 3 Vavilov Institute of General Genetics, Russian Academy of Sciences, 117971 Moscow, Russia; ivan.kulakovskiy@gmail.com; 4 Institute of Protein Research, Russian Academy of Sciences, 142290 Pushchino, Russia; 5 Laboratory of Regulatory Genomics, Institute of Fundamental Medicine and Biology, Kazan Federal University, 420008 Kazan, Russia

**Keywords:** translation factors, aminoacyl-tRNA synthetases ARSases, transcriptome, proteome, transcriptional landscape, organ-specific translation, cell type-specific translation, neurons, gonads, sexual dimorphism

## Abstract

While protein synthesis is vital for the majority of cell types of the human body, diversely differentiated cells require specific translation regulation. This suggests the specialization of translation machinery across tissues and organs. Using transcriptomic data from GTEx, FANTOM, and Gene Atlas, we systematically explored the abundance of transcripts encoding translation factors and aminoacyl-tRNA synthetases (ARSases) in human tissues. We revised a few known and identified several novel translation-related genes exhibiting strict tissue-specific expression. The proteins they encode include eEF1A1, eEF1A2, PABPC1L, PABPC3, eIF1B, eIF4E1B, eIF4ENIF1, and eIF5AL1. Furthermore, our analysis revealed a pervasive tissue-specific relative abundance of translation machinery components (e.g., PABP and eRF3 paralogs, eIF2B and eIF3 subunits, eIF5MPs, and some ARSases), suggesting presumptive variance in the composition of translation initiation, elongation, and termination complexes. These conclusions were largely confirmed by the analysis of proteomic data. Finally, we paid attention to sexual dimorphism in the repertoire of translation factors encoded in sex chromosomes (eIF1A, eIF2γ, and DDX3), and identified the testis and brain as organs with the most diverged expression of translation-associated genes.

## 1. Introduction

In multicellular organisms, differences between cell types are largely defined by specific patterns of gene expression. The cell specialization is partially determined at the translational level. Multiple studies showed that gene expression profiles at the level of transcription and translation vary across organs (see [1,2] and references therein). However, the differential regulation of protein synthesis across cell types and tissues is still poorly studied [3,4].

Recent advances in high-throughput approaches gave a new impetus to research in this field [5]. The cell type-specific expression of tagged ribosomal proteins provided a methodology for affinity purification of polysomal mRNAs from defined cell populations within a complex tissue [6,7,8]. These approaches were further enhanced by integration with ribosome profiling (Ribo-Seq), a technique that allows the analysis of ribosome occupancy of mRNA on a transcriptome-wide scale [9,10]. Although these techniques enabled quantitative and qualitative characterization of translatome from specific cells and lineages [10,11], existing studies have a limited ability to assess a bona fide translation efficiency of particular transcripts due to a lack of the complementing transcriptome information (RNA-Seq) from the same cells. Recently, studies involving Ribo-Seq of animal tissues and organs revealed organ-specific differences in translation efficiency and elongation speed [12,13,14,15]. Other findings revealed different tRNA repertoires and codon usage in different tissues [16,17], as well as peculiar features such as the readthrough rate [18]. However, to which extent the tissue-specificity of gene expression is determined at the translation level is still a poorly resolved issue.

It is clear that the variability of the transcriptome across cell types requires specialization of the translation apparatus. Although protein synthesis machinery is indispensable for life and hence thought to be ubiquitously present, recent systematical analyses revealed that some ribosomal components [19,20,21] and tRNAs [22,23] demonstrate tissue-specific expressions. This is likely true for other components of translation machinery, such as translation factors, aminoacyl-tRNA synthetases (ARSases), and other auxiliary proteins, but only a few cases of the tissue-specific expression of these components have been described until now.

The classic example is the well-documented tissue-specific expression of two eEF1A paralogs in rodents, with eEF1A2 present only in the brain, heart, and muscle and eEF1A1 expressed in all other tissues [24,25]. Testis-restricted transcription was reported for the human *PABPC3* gene [26], which is absent in mice, while gonad-specific expression of other poly(A)-binding protein paralog, PABPC1L/ePAB, is controlled at both transcriptional and post-transcriptional levels in mice and humans [27,28]. An elevated concentration in the testis was also shown for translation elongation factor eIF5A2 [29]. Diverged cap-binding protein eIF4E1B was declared to be oocyte-specific in some vertebrates (for review, see [30]), although its expression has not been extensively analyzed across multiple tissues and organs.

The above fragmentary information indicates that the expression of selected translation machinery components is restricted to particular cell types or tissues. It is even more intriguing that the abundance of canonical translation factors, such as eIF2γ, eIF5, ETF1/eRF1, GSPT2/eRF3b, eEF1B subunits, or mitochondrial translation factors, also varies significantly across tissues [31,32,33,34,35,36,37,38,39,40]. Thus, the uniformity of translation apparatus in different tissues is illusory, suggesting that distinct translational properties of human cell types exist that provide an additional layer of tissue-specific regulation of gene expression.

As mammalian organs require diverse levels of protein synthesis activity [41,42,43], it is expected that some of the tissues have more translation machinery components, including ribosomes, than others [19,20,44]. However, it remains unclear whether this difference significantly affects translation regulation, as, in many cases, it is determined not by the absolute but by the relative abundance of translation factors [45]. To our knowledge, no such analyses have been performed yet.

Here, using gene expression data from multiple sources, we systematically analyzed the abundance of mRNAs encoding translation factors and ARSases across human tissues. We found intriguing differences in the transcript levels, not only in terms of the absolute values but also in relative shares within physically and functionally related complexes. The diverged expression repertoire of translation-associated genes was especially prominent in the testis and brain. Where possible, we confirmed these observations by analysis of proteomic data.

## 2. Results

### 2.1. Characterization of a Tissue-Specific Expression Pattern of Two Human eEF1A Paralogs Using an Integrated Transcriptomic and Proteomic Data Analysis

We analyzed transcriptomic data from GTEx, FANTOM, and Gene Atlas databases, available for various human tissues, to assess the differential expression of genes encoding translation factors, ARSases, and auxiliary proteins (hereafter called TAG, for translation-associated genes). Since different cell types clearly have distinct requirements for protein synthesis and its efficiency, expected levels of translation machinery components vary in a wide range. This has previously been shown for the expression of ribosomal protein genes [19,20] (see also our rough estimate of their mean transcript abundance in GTEx shown in Supplementary Figure S1).

Therefore, to access the differences in the composition of translation machinery across tissues, we analyzed the relative abundance of the TAG transcripts within functional complexes. To this end, we determined several complexes and functional groups of translation-associated proteins (Table 1). TAGs were combined into groups based on known physical and functional interactions of their products. Each factor could be included in more than one group, and selected smaller groups were fully included in the larger ones. Importantly, the total expression of genes encoding components of the large groups such as “INITIATION”, “ELONGATION”, “TERMINATION”, or “ARSases” is well correlated with that of ribosomal protein genes (with a few exceptions, such as ovary in all groups, or brain and muscle in “ELONGATION”), as shown in Supplementary Figure S2. Next, we calculated two parameters for each gene, the transcript abundance value (see Materials and Methods) and the relative abundance of the transcript within the group. The latter allowed us to detect putative differences in the stoichiometry of the complexes.

The classic example of TAGs with a highly pronounced tissue-specific expression is the genes encoding rodent eEF1A paralogs. The translation elongation factor eEF1A delivers aminoacyl-tRNA to the ribosomal A-site for decoding, which is critical for protein synthesis [46,47]. It is also one of the most abundant proteins in the cell. In many eukaryotes, eEF1A is encoded by two paralogous genes. In mice and rats, these genes (*Eef1a1* and *Eef1a2*) have a well-documented mutually exclusive expression pattern [25,48]. Here, we used the example of their human orthologs, *EEF1A1*, and *EEF1A2*, to illustrate a strategy for the characterization of tissue-specific TAG expression.

**Figure 1:**
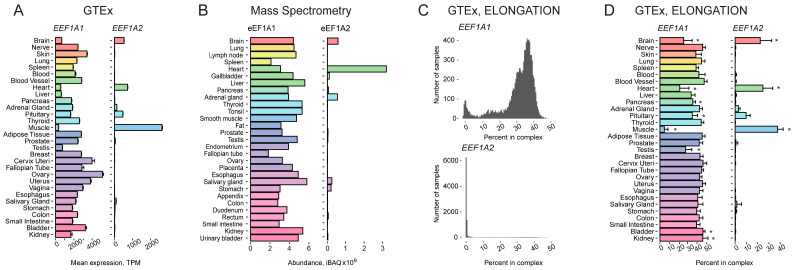
Tissue-specific expression pattern of two human genes encoding eEF1A paralogs, *EEF1A1* and *EEF1A2*. (**A**) Expression of the *EEF1A1* and *EEF1A2* genes in various human tissues according to GTEx. (**B**) Levels of eEF1A1 and eEF1A2 proteins in various human tissues according to high-throughput proteomic analysis [1]. (**C**) Histograms showing the distribution of the percentage of *EEF1A1* (top) and *EEF1A2* (bottom) expressions among the genes from the “ELONGATION” complex across various human tissues, according to GTEx. (**D**) Percentage of *EEF1A1* and *EEF1A2* expressions among the genes from the “ELONGATION” complex across various human tissues, according to GTEx. TPM, Transcripts Per Kilobase Million; *, FDR corrected *p*-value < 0.01 in enrichment analysis (fgsea R package).

Analysis of GTEx data showed that human *EEF1A2* is exclusively expressed not only in the brain, heart, and muscle (similarly to the rodent gene) but also in the pituitary and, to a lesser extent, in adrenal and salivary glands (Figure 1A). The expression pattern of *EEF1A2* was in agreement with proteomics data from one of the most representative proteomics atlas of human tissues (Figure 1B) [1]. Notably, the gland-specific expression of this gene has not been documented before.

As expected, the expression of *EEF1A1* generally mirrored that of *EEF1A2*, being reduced in the organs and tissues with elevated *EEF1A2* expression (Figure 1A,B). However, a closer examination of its mRNA and protein representation among components of the “ELONGATION” complex revealed that, while relative eEF1A1 abundance was strongly reduced in skeletal muscle in comparison with most other tissues, in heart and brain samples, it remained at a high level, indicating the simultaneous presence of two eEF1A paralogs in these samples (Figure 1C,D). This contradicts the widely accepted view that the two eEF1A variants are mutually exclusive in tissues. Similar results were obtained with data from Gene Atlas and FANTOM databases (Supplementary Figure S3A,B). Due to a slightly different set of organs and tissues analyzed in the three projects, *EEF1A2* expression in the tongue, eye, throat, and pineal gland also became evident.

To further strengthen our conclusions, we also used an alternative normalization approach. We took the weighted total of the ribosomal protein gene expression (see above, Supplementary Figure S1) instead of the “ELONGATION” complex genes to normalize the abundance of the *EEF1A1* and *EEF1A2* transcripts and obtained essentially the same results (Supplementary Figure S3C).

To additionally validate our method, we similarly processed the transcriptomic data for mRNAs encoding three subunits of eEF1B, a guanidine nucleotide exchange factor for eEF1A [47]: *EEF1B2*/eEF1Bα, *EEF1D*/eEF1Bδ, and *EEF1G*/eEF1Bγ. It was shown previously that absolute abundances of eEF1B subunits vary significantly across mammalian tissues [37]. In accordance with this, we found that the mRNA levels differ greatly between human tissues (Supplementary Figure S4A); however, their relative amounts showed a much more even distribution (Supplementary Figure S4B), suggesting a coordinated expression of the three genes and consistent with the essential function of the eEF1B complex in translation. A similar relative accordance is observed at the protein level (Supplementary Figure S4C,D), although with some outliers explained either by a lower representation of mass-spectrometry data or by post-transcriptional regulation.

In mammalian cells, eEF1A, together with eEF1B and valyl-tRNA synthetase (VARS1), forms a supercomplex called eEF1H (for review, see [47]). Thus, we also analyzed the relative abundance of eEF1H components at both mRNA and protein levels (Supplementary Figure S5). This analysis revealed an intriguingly high relative abundance of mRNA encoding VARS1 in the testis that was likely neutralized at the post-transcriptional level.

### 2.2. Tissue Specificity of Translation-Associated Proteins Encoded in Sex Chromosomes

To further validate our approach, we assessed the tissue specificity of translation-associated proteins encoded by sex chromosomes. Obviously, the expression of Y-encoded TAGs should not be detected in female-specific tissues. Among 65 protein-encoding genes located at the human Y chromosome [49], there are only two TAGs from our list, *EIF1AY* and *DDX3Y*. Both of them have paralogs at the X chromosome, *EIF1AX* and *DDX3X*, respectively.

**Figure 2:**
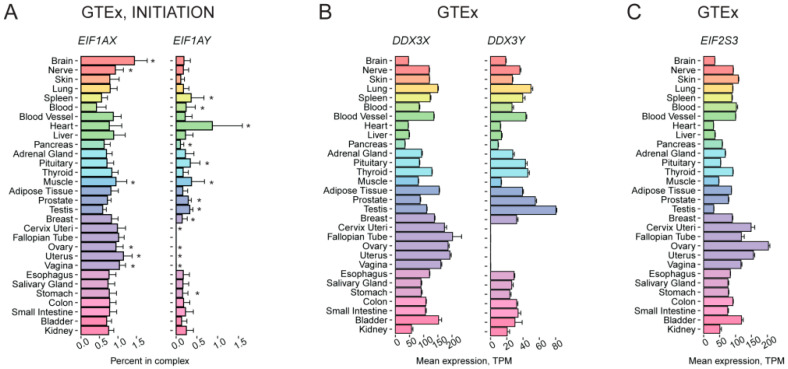
Tissue-specific expression pattern of translation-associated genes localized in sex chromosomes. (**A**) Percentage of *EIF1AX* and *EIF1AY* expression among the genes from the “INITIATION” complex across various human tissues, according to GTEx. (**B**) Expression of the *DDX3X* and *DDX3Y* genes in various human tissues according to GTEx. (**C**) Expression of the *EIF2S3* gene in various human tissues according to GTEx. TPM, Transcripts Per Kilobase Million; *, FDR corrected *p*-value < 0.01 in enrichment analysis (fgsea R package).

*EIF1AX* and *EIF1AY* are paralogs coding for the canonical translation initiation factor eIF1A, which is indispensable for 48S preinitiation complex formation [45]. Two eIF1A variants are almost identical (they differ by only one amino acid out of one hundred forty-four, Leu50 in eIF1AY and Met50 in eIF1AX in humans) and thus probably have identical functions. An equal overall eIF1A level in male and female cells thus requires that the *EIF1AX* gene escapes X chromosome inactivation, which is indeed the case [50,51]. Analysis of *EIF1AX* and *EIF1AY* mRNA abundance confirmed the absence of the *EIF1AY* transcript in female-specific organs and revealed the differential expression between tissues (Supplementary Figure S6A). We then calculated their relative abundance within the group “INITIATION”. This approach revealed a slightly variable relative abundance of the *EIF1AX* mRNA, while the expression level of the *EIF1AY* gene was not uniform (Figure 2A). This analysis also showed a predominance of the *EIF1AX* gene as a source of the eIF1A protein but pointed to the heart as an organ with an apparently higher overall eIF1A level due to the elevated impact of *EIF1AY* expression. The proteomic data [1] available only for eIF1AX revealed its depletion in the testis in comparison with other factors of the “INITIATION” complex (Supplementary Figure S6B). This was not seen in transcriptomic data from either source and suggested an additional layer of regulation at the post-transcriptional level, as well as a putative role in specialized translational control in this organ. Interestingly, the presence of the Y-encoded eIF1A isoform is not a conserved feature in mammals, as mice have only one gene, *Eif1ax*, which does not escape X inactivation [52].

*DDX3X* and *DDX3Y* produce isoforms of DEAD-box helicase, DDX3, an auxiliary translation initiation component, which plays an essential role in eukaryotic RNA metabolism [53]. The two proteins share only 92% identity. *DDX3X* cannot substitute for *DDX3Y* function, while the replacement of *DDX3X* with *DDX3Y* does not affect the translation rate [54,55,56]. Deletions encompassing the *DDX3Y* gene are thought to result in spermatogenic failure (reviewed in [57,58]), although this opinion has been challenged recently [59]. Mutations in *DDX3X* are a cause of intellectual disability in females and a decreased viability in males (reviewed in [60]), which, as Venkataramanan et al. suggest, can be explained by the tissue-specific expression of *DDX3X* and *DDX3Y* [56]. Similar to *EIF1AX*, *DDX3X* escapes X-chromosome inactivation [52].

Our data analysis showed the absence of *DDX3Y* mRNA and protein in female-specific tissues that met our expectations. In contrast, both the mRNA and protein levels were high in the testis. *DDX3X* is expressed ubiquitously; however, the abundance of its transcript is somewhat higher in the female reproductive system (Figure 2B), but this slight difference is not reflected in proteomics data (Supplementary Figure S6C).

In humans, X-chromosome contains one more unevenly expressed TAG—*EIF2S3*. It codes for the γ-subunit of eIF2, a critical translation machinery component required for Met-tRNA_i_ delivery during translation initiation. GTEx data analysis revealed that the *EIF2S3* transcript abundance is higher in female reproductive tissues than in testes (Figure 2C), which is in agreement with its X-inactivation escape profile [61]. The relative expression of eIF2 complex components (Supplementary Figure S6D) varies between female and male reproductive tissues accordingly. Interestingly, mice have a paralog of *Eif2s3x*, *Eif2s3y*, which is required to drive spermatogenesis and represents one of the two genes irreplaceable for male mice fertility [62]. Mouse *Eif2s3x* escapes from X inactivation similarly to the human gene [52,61] and is expressed higher in female mice than in males in developing and adult brains at the transcriptional level [31]. Notably, mutations in the human *EIF2S3* result in the brain affecting MEHMO syndrome (Mental retardation, Epileptic seizures, Hypogenitalism, Microcephaly, and Obesity) in males [63].

### 2.3. A Number of Translation Factors Have a Pronounced Tissue-Specific Representation within Corresponding Functional Groups

*PABPC1* is usually considered the main source of cytoplasmic poly(A)-binding proteins in cells; however, our analysis indicates that some tissues have specifically produced PABP paralogs, which may act together with PABPC1 or partially substitute it. Not all of them were represented in proteomic datasets, but the available information, together with transcriptomic data, is sufficient to draw the following conclusions.

**Figure 3:**
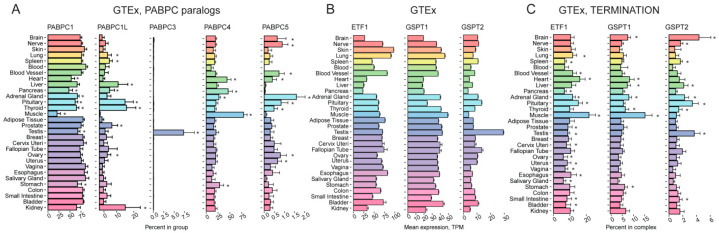
Tissue-specific expression pattern of genes encoding PABPC homologs and translation termination factors. (**A**) Percentage of *PABPC1*, *PABPC1L*, *PABPC3*, *PABPC4*, and *PABPC5* expression among the genes from the “PABPC” complex across various human tissues, according to GTEx. (**B**) Expression of the *ETF1*, *GSPT1*, and *GSPT1* genes encoding eRF1, eRF3a/GSPT1, and eRF3b/GSPT2 correspondingly in various human tissues according to GTEx. (**C**) Percentage of *ETF1*, *GSPT1*, and *GSPT1* expression among the genes from the “TERMINATION” complex across various human tissues, according to GTEx. TPM, Transcripts Per Kilobase Million; *, FDR corrected *p*-value < 0.01 in enrichment analysis (fgsea R package).

The level of the *PABPC1* transcript is relatively high in reproductive tissues (Supplementary Figure S7A), while it is reduced in brain, heart, and skeletal muscles. The expression pattern is reflected in the “INITIATION” and, to some extent, “TERMINATION+” complexes (Supplementary Figure S7B). Analysis of the relative *PABPC1* mRNA abundance among all PABPC paralogs (hereafter called “PABPC paralogs” group) also revealed a significant decline of its expression in muscles (Figure 3A), suggesting that its role in this organ may be of lower importance.

*PABPC1L*/*ePAB* was previously reported to have a gonad- and embryo-restricted expression [26,27]. This is consistent with its role in oocyte maturation in mice and its importance for female fertility in both mice and humans [64,65]. However, our analysis also unexpectedly revealed a high level of this transcript in the pituitary and thyroid glands, as well as in the liver and kidney, relative to that of other components of the “PABPCs complex” (Figure 3A). Its putative function in hormone-producing organs can contribute to its role in fertility.

PABPC3 was previously shown to be abundant only in the testis [26]. Our analysis confirmed this highly specific expression pattern at the mRNA level (Figure 3A), but the protein level was unexpectedly high not only in the testis but also in adipose tissue, colon, and prostate (Supplementary Figure S7C). This case highlights the importance of addressing gene expression regulation at different levels. Importantly, *PABPC1*, *PABPC1L*, and *PABPC3* expressions are perturbed in infertile men [66].

PABPC4 is able to compensate for the loss of PABPC1 [67]. In accordance with this, our approach revealed that *PABPC4* is upregulated at the mRNA and protein levels in muscle, heart, and pancreas in the “PABPCs complex”, where *PABPC1* and *PABPC5* are downregulated (Figure 3A).

PABPC5 is an X-linked poly(A)-binding protein [68]. The analysis of its relative abundance within “PABPC paralogs” revealed a relatively decreased level of the *PABPC5* transcript in the pancreas, muscle, liver, salivary gland, and blood (Figure 3A, Supplementary Figure S7D). Interestingly, the pancreas, muscle, and liver are among the most translationally active tissues containing the highest amount of ribosomes [44], so this pattern suggests its regulatory role in general protein synthesis.

eRF1 is an essential translation termination factor encoded by the *ETF1* gene. *ETF1* was claimed to be expressed at a high level in the testis, brain, heart, and kidney and downregulated in the liver and colon [32]. The data obtained from GTEx (Figure 3B) only partially confirmed this, indicating the *ETF1* mRNA abundance is lower in the liver, pancreas, kidney, and heart (likely the most metabolically active tissues [69]), as well as in the brain, while there no significant difference between the colon and other tissues was observed. Remarkably, *ETF1*, while encoding a general translation factor, demonstrated an uneven transcription level within the “TERMINATION+” group: its expression is higher in the muscle, heart, and pituitary (Figure 3C).

*GSPT1* and *GSPT2* code for GTP-binding termination factors, eRF3a/GSPT1 and eRF3b/GSPT2, respectively [35]. The factors have at least partially redundant functions, as eRF3b can compensate for a silenced eRF3a [36]. We found that the level of *GSPT1* mRNA quite closely corresponds to that of mRNA encoding eRF1 (Figure 3B,C). On the contrary, analysis of the *GSPT2* mRNA abundance suggests its specific relative increase in the brain and testis in comparison to other “TERMINATION” components (Figure 3C). The elevated *GSPT2* mRNA level in the brain is consistent with Hoshino results [35] and may reflect a substituting role of eRF3b in non-proliferating cells, where eRF3a should be depleted.

**Figure 4:**
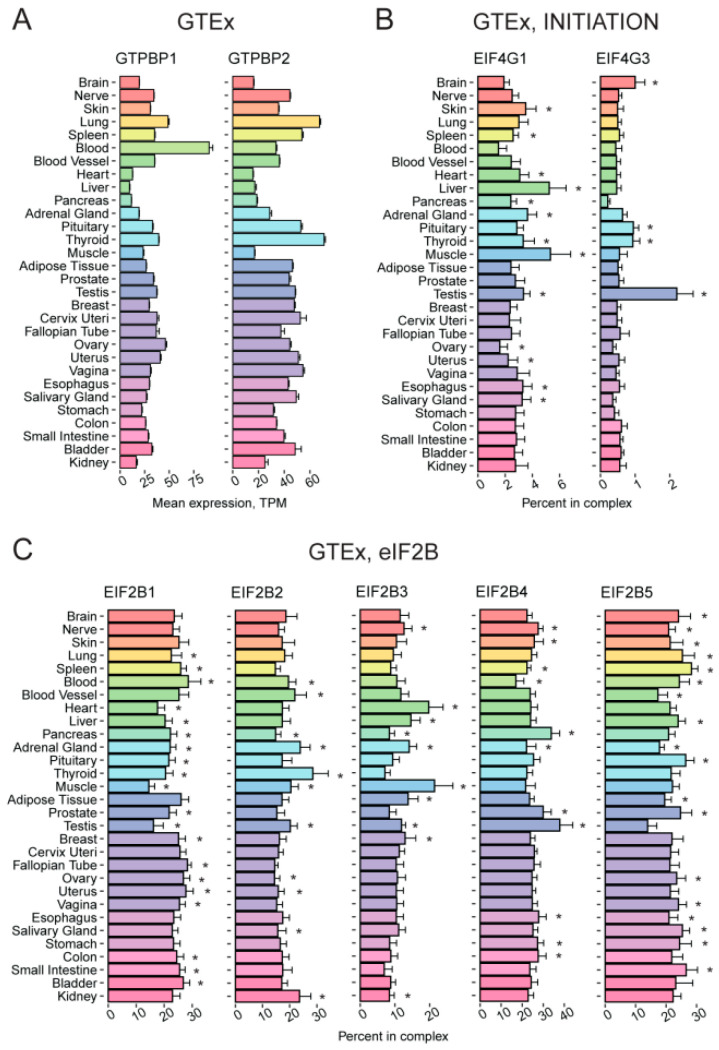
Tissue-specific expression pattern of genes encoding GTPBP1 and GTPBP2 proteins, eIF4G paralogs, and eIF2B subunits. (**A**) Expression of the *GTPBP1* and *GTPBP2* genes in various human tissues according to GTEx. (**B**) Percentage of *EIF4G1* and *EIF4G3* among the genes from the “INITIATION” complex across various human tissues, according to GTEx. (**C**) Percentage of *EIF2B1*, *EIF2B2*, *EIF2B3*, *EIF2B4*, and *EIF2B5* expression among the genes encoding components of the “eIF2B” complex across various human tissues, according to GTEx. TPM, Transcripts Per Kilobase Million; *, FDR corrected *p*-value < 0.01 in enrichment analysis (fgsea R package).

GTPBP1 and GTPBP2 are GTPases involved in translational control: GTPBP1 has eEF1A-like elongation activity, while GTPBP2 is likely involved in stalling ribosome rescue [70,71]. Their expression in mammalian tissues is poorly studied. The mouse brain was shown to have an elevated level of the *GTPBP1* transcripts [72], while the testis and thymus are the organs with an increased abundance of the *GTPBP2* mRNA [73]. Our analysis of GTEx data revealed blood as a tissue with the highest level of the *GTPBP1* mRNA in the human body (Figure 4A), suggesting their specific role in blood cell differentiation and physiology. Unfortunately, these tissues were not represented in the proteomics data that we analyzed in this study. 

*EIF4G3* codes for a paralog of eIF4G1. Both eIF4G1 and eIF4G3 are able to bind cap-binding protein eIF4E and RNA helicase eIF4A, thus forming the trimeric translation initiation factor eIF4F. In humans, these paralogs share less than 50% identity and could have slightly different functions [74]. It was demonstrated previously that *EIF4G1* is highly expressed in the liver and testis, while *EIF4G3* is upregulated in the testis and fetal brain and downregulated in the lung, heart, liver, and placenta [75,76]. In agreement with these data, we found the level of *EIF4G3* transcript is elevated in the testis (Supplementary Figure S8A) or in the testis and brain if considered within the “INITIATION” group (Figure 4B). This is consistent with the fact that *EIF4G3* mutations cause male infertility [77]. Proteomic data confirmed the elevated level of eIF4G3 in the testis and additionally revealed its elevated abundance in the small intestine and smooth muscle (Supplementary Figure S8B). Thus, while eIF4G1 likely represents the major source of eIF4G activity in most tissues (Figure 4B, Supplementary Figure S8B), eIF4G3 might differentially contribute to translation in different organs, providing a fine-tuning of cap-dependent and alternative translation initiation pathways [78].

eIF2B is a guanine nucleotide exchange factor for eIF2. It consists of five subunits, α to ε, encoded by *EIF2B1*-*EIF2B5* genes, all of which are linked to a severe inherited human neurodegenerative disorder called Leukoencephalopathy with Vanishing White Matter, or VWM (for review, see [79]). Intriguingly, eIF2B subunits are known to form a number of differentially composed sub-complexes with non-identical activity [79]. Thus, we performed a comprehensive analysis of the relative abundance of individual eIF2B subunits and their mRNAs in comparison to those of all the subunits (the “eIF2B” group). At the mRNA level, we revealed the ubiquitous expression of five genes with no more than ~2-fold difference between tissues (Figure 4C), probably with some deviations in the testis (with a lower *EIF2B1* and *EIF2B5* levels and a higher *EIF2B4* one), muscles, and heart (lower *EIF2B1* and higher *EIF2B3* levels). Importantly, we observed no prominent specificity in subunit abundance either at the mRNA or protein level in the brain, suggesting that the neural manifestation of VWM is not related to a distinct eIF2B composition in this organ. Mass-spectrometry data showed a more uneven subunit distribution (Supplementary Figure S8C), with putatively distinct stoichiometry in organs: for example, in the heart, liver, thyroid, salivary gland, small intestine, and kidney, EIF2B4 and EIF2B5 have an abundance above average levels, while EIF2B1 is below the average; in contrast, in the gallbladder, pancreas, endometrium, and esophagus, the proportions seem to be the opposite. While the discrepancy at the mRNA and protein levels can be partially explained by a lower quality of the mass-spectrometry data, it should be noted that the abundance of eIF2B subunits is known to be regulated at the post-transcriptional level [80]. Overall, these findings suggest a diverse composition of the eIF2B complex across the human tissues.

Another multisubunit initiation factor is eIF3. Depending on the organism and terminology, up to 13 proteins can be considered its subunits, named eIF3a-eIF3m (Table 1) [81,82,83]. Although some eIF3 subunits are thought to form sub-complexes or even function as independent proteins [83], our analysis revealed no cases of prominent tissue-specific expression of any of the subunits (Supplementary Figure S9). However, some fluctuations can be observed, for example, in the abundance of the *EIF3G* (higher in the testis), *EIF3J* (lower in the ovary and higher in the brain, liver, and muscles), and *EIF3K* (higher in the heart) transcripts.

### 2.4. New Striking Examples of Tissue-Specific Expression of Genes Encoding Translation Factors

**Figure 5:**
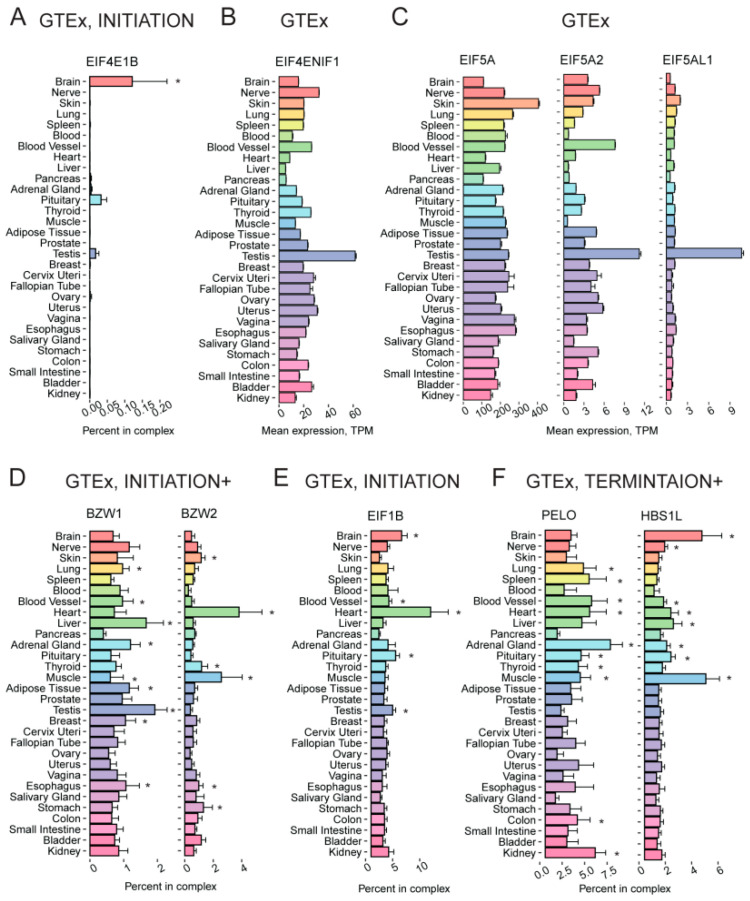
Tissue-specific expression pattern of translation-associated genes showing high tissue specificity. (**A**) Percentage of *EIF4E1B* expression among the genes from the “INITIATION” complex across various human tissues, according to GTEx. (**B**) Expression of the *EIF4ENIF1* in various human tissues according to GTEx. (**C**) Expression of the *EIF5A*, *EIF5A2*, and *EIF5AL1* in various human tissues according to GTEx. (**D**) Percentage of *BZW1* and *BZW2* expression among the genes from the “INITIATION+” complex across various human tissues according to GTEx. (**E**) Percentage of *EIF1B* expression among the genes from the “INITIATION” complex across various human tissues, according to GTEx. (**F**) Percentage of *PELO* and *HBS1L* expression among the genes from the “TERMINATION+” complex across various human tissues according to GTEx. TPM, Transcripts Per Kilobase Million; *, FDR corrected *p*-value < 0.01 in enrichment analysis (fgsea R package).

Translation initiation factor eIF4E1B belongs to the eIF4E cap-binding protein family, but it has a low affinity for the m^7^G-cap [84]. In amphibians, the eIF4E1B orthologue is part of a multisubunit complex that specifically inhibits the translation of some mRNAs in oocytes [84,85]. Thus, it has been proposed that *EIF4E1B* expression is limited to ovaries and oocytes, where the factor plays a role in the translational repression of maternal mRNAs ensures subsequent reprogramming of the zygotic genome [84,86], while its canonical ortholog *EIF4E1* is transcribed ubiquitously to support the cap-dependent translation. Unexpectedly, besides the ovary, we found a high level of the *EIF4E1B* expression also in the testis, retina, spinal cord, and brain, including pineal and pituitary glands (Figure 5A, Supplementary Figure S10A). This peculiar pattern of expression is probably related to that well documented for its putative partner CPEB [85,87]. 

*EIF4ENIF1* encodes a multifunctional eIF4E-binding protein 4E-T that plays a role in the nucleocytoplasmic shuttling of eIF4E and in translational repression [88,89]. Interestingly, it binds not only the canonical eIF4E protein but also its orthologs (including eIF4E1B). Our analysis revealed that testis has the highest level of *EIF4ENIF1* transcript and protein (Figure 5B, Supplementary Figure S10B,C).

eIF5A, a translation elongation factor having the unique post-translation modification hypusine, is encoded in the human genome by three genes: *EIF5A*, *EIF5A2*, and *EIF5AL1*. eIF5A1 (encoded by *EIF5A*) and EIF5AL1 are almost identical (98%), while eIF5A2 is slightly more diverged (84% identity) [29]. eIF5A1 is thought to be ubiquitous, while the *EIF5A2* expression has been reported to be restricted by brain and testis only, where it is represented by two or more isoforms with different 3′ UTR lengths [29,90]. Our analysis of GTEx and mass-spectrometry data confirmed the ubiquitous expression of *EIF5A* but revealed a more complex pattern of the *EIF5A2* expression (Figure 5C, Supplementary Figure S10D). In particular, eIF5A2 relative abundance is much lower at both mRNA and protein levels in the liver and probably some other visceral organs but noticeably higher in the testis. Additionally, we revealed a strict testis-specific expression of *EIF5AL1*: according to GTEx, this gene is almost exclusively expressed in this organ (Figure 5C). Yet, there was no information about the protein level of EIF5AL1. Taking into account the high similarity of eIF5A paralogs, it can be assumed that protein synthesis in male gonads specifically requires a higher eIF5A concentration, although a distinct testis-specific function of eIF5AL1 cannot be ruled out either.

BZW1/5MP2 and BZW2/5MP1 are eIF5-mimic proteins that regulate the stringency of start site selection [91]. The relative level of *BZW1* mRNA varies moderately between tissues, being highest in the liver and testis and lowest in the pancreas (Figure 5D). In contrast, the relative abundance of *BZW2* transcript (which is overall lower if assessed in the context of the “INITIATION+” group) is noticeably high in only two tissues, the heart and muscle (Figure 5D). 

The elevated expression of *BZW2* in the heart is also evident at the protein level, and it is also high in the placenta (Supplementary Figure S10E). This suggests that BZW1/5MP2 is likely a general regulator of protein synthesis, while BZW2/5MP1 could be a specialized factor for tissue- and mRNA-specific translational control.

A poorly studied protein, eIF1B is 92% identical to its paralog eIF1, which plays a major role in the start codon selection along with eIF5 [92]. While *EIF1* relative expression is ubiquitous with little variations across organs (Figure 5E), *EIF1B* mRNA relative abundance is clearly higher in the heart and brain while lower in the skin and pancreas (Figure 5E). An even more complex distribution of eIF1B across tissues is observed at the protein level (Supplementary Figure S10F), in agreement with a proposed regulation at the level of translation [93]. The high similarity between eIF1B and eIF1, as well as some indirect data (see [92] and references therein), suggests the functional redundancy of these proteins. Thus, an increased total amount of the eIF1/eIF1B activity could contribute to tissue-specific regulation of initiation codon selection similar to that observed in cells artificially overexpressing eIF1 [94].

We also detected tissue-specific expression of some translation quality control factors that had not been reported before. *PELO* and *HBS1L* encode non-canonical termination factors mediating the dissociation of inactive, vacant, or stalled ribosomes (for review, see [95]). At least one case of a cell-type specific translational control by PELO and HBS1L abundance has been reported [96]. It is also important to note that HBS1L deficiency causes organ-specific defects during development [97]. These facts suggest the involvement of the factors in tissue-specific regulation of gene expression. Although PELO and HBS1L are thought to work in tandem, analyses of transcriptomic and mass-spectrometry data suggest that their abundances do not correlate well across tissues. In particular, we would like to report the inverse ratio of the encoding mRNAs in the brain and muscle (where the relative level of *HBS1L* mRNA is higher than that of *PELO*) vs. the adrenal gland, kidney, and spleen (where the proportion is inverse), see Figure 5F. At the protein level, the liver, gallbladder, duodenum, and testis have relatively higher levels of HBS1L than PELO, while the thyroid, prostate, and bladder demonstrate the opposite tendency (Supplementary Figure S10G).

### 2.5. Genes Encoding Some ARSases Also Have a Pronounced Tissue-Specific Expression

Aminoacyl-tRNA synthetases (ARSases) are key components of protein synthesis machinery. Surprisingly, we found that some of them also have diverse expression patterns across organs and tissues. As before, we analyzed absolute and relative mRNA and protein abundance of ARSases (including mitochondrial and dual-targeted ones) within functional groups. In Metazoa, some ARSases form a multiprotein complex with auxiliary factors, called Multiple ARSase Complex (MARS) [98]. Thus, we used two functional groups: “ARSases” and “ARSase COMPLEX” (Table 1).

**Figure 6:**
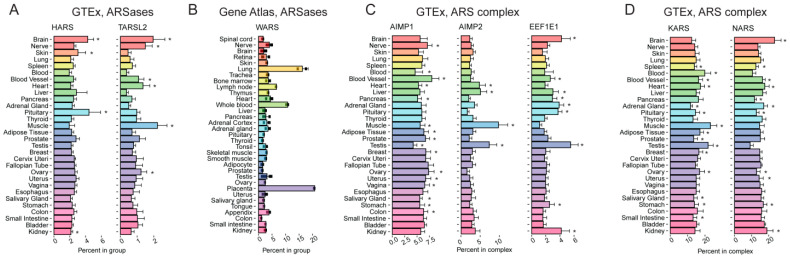
Tissue-specific expression pattern of genes encoding several aminoacyl-tRNA-synthetases (ARSases) showing tissue specificity. (**A**) Percentage of *HARS* and *TARSL2* expression among the genes from the “ARSases” complex across various human tissues, according to GTEx. (**B**) Percentage of *WARS* expression among the genes from the “ARSases” complex across various human tissues, according to Gene Atlas [101]. (**C**) Percentage of *AIMP1*, *AIMP2*, and *EEF1E1* expression among the genes from the “ARSase COMPLEX” complex across various human tissues, according to GTEx. (**D**) Percentage of *KARS* and *NARS* expression among the genes from the “ARSase COMPLEX” complex across various human tissues, according to GTEx. *, FDR corrected *p*-value < 0.01 in enrichment analysis (fgsea R package).

*HARS1* gene encoding histidyl-tRNA synthetase (*HARS1*) has been previously reported as highly expressed in the heart, brain, liver, and kidney [99]. According to our analysis, the corresponding mRNA is relatively more abundant in the brain, especially in the pituitary, as compared to other ARSases, although more or less homogenously distributed in other organs (Figure 6A, Supplementary Figure S11A). The *TARSL2*/*TARS3* gene encoding threonyl-tRNA synthetase is relatively upregulated in the brain, spinal cord, muscle, and heart. The data from GTEx and FANTOM revealed that *TARSL2* was efficiently transcribed in the brain and muscles (Figure 6A, Supplementary Figure S11A). It is consistent with its high abundance in muscle and heart in mice, as reported earlier [100]. Lung, blood, and placenta are the organs with increased relative levels of tryptophanyl-tRNA synthetase (*WARS*) transcript and protein (Figure 6B, Supplementary Figure S11B).

*AIMP2* codes for a scaffold protein of the MARS complex, AIMP2, which has two partners: AIMP1 and EEFE1/AIMP3 [98]. The data obtained from all three databases indicated that the relative abundance of *AIMP2* transcript (as compared to that of all “ARSase COMPLEX” components, see Table 1) is elevated in muscle tissues and testis (Figure 6C). In contrast, *AIMP1* mRNA and protein did not show any prominent tissue-specific pattern (Figure 6C and Supplementary Figure S11C), The third key MARS component, EEF1E1, shows no unambiguous correlation between mRNA and protein levels, suggesting regulation of its abundance at the translational level.

ARSase components of MARS can also be differentially distributed across tissues. A more or less homogenous expression is exemplified by the *KARS* gene coding for lysyl-tRNA synthetase (Figure 6D and Supplementary Figure S11D). In contrast, the relative expression of *NARS* (encoding asparaginyl-tRNA synthetase), although similarly high in most tissues, is further elevated in the brain but repressed in the testis, as evident at both mRNA and protein levels (Figure 6D and Supplementary Figure S11D). Thus, MARS composition may vary in different human organs and tissues.

## 3. Discussion

mRNA translation is one of the basic processes in a living cell. However, it is clear that in a multicellular organism, various cell types differentially rely on the synthesis of new proteins and thus require different amounts of translation machinery components. In addition, the composition of this apparatus can contribute to the translational control of gene expression. Thus, human tissues and organs may have diverse concentrations, ratios, and architectures of ribosomes and ribosomal complexes, tRNAs, translation factors, and other auxiliary proteins.

Differential abundance of tRNA species in human tissues was reported previously [22,23]. Expression levels of genes coding for ribosomal proteins and rRNAs were found to vary significantly across human tissues as well [20,44,102,103]. A concept of the specialized ribosomes (comprehensively reviewed in [104]) even states that different mRNA species can be differentially translated by heterogeneously composed ribosomal particles, depending on cell types and conditions. The variable composition of ribosomes has indeed been confirmed by a number of studies [19,20,21,105]. Interest in this area has especially increased in recent years thanks to the research by Maria Barna and colleagues [106].

Here, we present evidence that many translation factors, both general and non-canonical ones, as well as auxiliary proteins such as ARSases, also show tissue-specific expression patterns, dictating diverse composition of translation apparatus in different cell types. In particular, we systematically explored three transcriptomic databases (GTEx, FANTOM, and Gene Atlas) and one source of proteomic data [1] and found a number of translation machinery components with strict tissue-specific appearance: eEF1A1, eEF1A2, PABPC1L, PABPC3, eIF1B, eIF4E1B, eIF4ENIF1, and eIF5AL1; we then identified a differential relative abundance of PABP and eRF3 paralogs, eIF2B and eIF3 subunits, eIF5-mimic proteins, and some ARSases, suggesting that even some general factors may have tissue-specific functions. We also noted sexual dimorphism in the repertoire of translation-associated proteins encoded in sex chromosomes: eIF1A, eIF2γ, and DDX3.

Recently, a global analysis of translation factors and tRNA expressions in human cancers was performed [107]. The authors revealed the overproduction of tRNA modification enzymes, ARSases, and other translation-associated proteins, which may play a role in the activation of protein synthesis across multiple cancer types. However, cancer cells are well known to have deregulated gene expression, so it was very important to show that normal human tissues can have a diverse composition of translation machinery as well.

Obviously, normal mammalian tissues have highly variable levels of protein synthesis, depending on metabolic rate, secretory activity, and cell proliferation status. A number of studies assessed protein synthesis rates across tissues by metabolic labeling [41,42,43]. These analyses revealed the highest amino acid incorporation and/or protein turnover in the small intestine and pancreas; intermediate in the kidney, spleen, and liver; and the lowest in lung, heart, brain, muscle, and adipose tissue. It was also shown that the protein synthesis rate is tightly coupled to cell metabolic fluxes [108]. Metabolic activities of mammalian tissues vary significantly, in a row, from heart and kidney (the most active ones) to skeletal muscle and adipose tissue [69]. Ribosome amounts also vary dramatically (∼50-fold) between the tissues, with more ribosomes present in the pancreas, salivary gland, liver, and intestine; medium in muscle, ovary, kidney, and brain cortex; and lower in the thymus, spleen, heart, lung, and cerebellum [44]. All this makes it clear that for the assessment of tissue-specific features of translation machinery, one should not use absolute values of mRNA or protein abundances of its components. Thus, in our analyses, we use a novel methodology when we combine translation factors into functional groups and calculate the relative mRNA or protein abundance of the particular component within the group. Importantly, we found a good correlation between expression levels of these functional groups (e.g., consolidated sets of initiation, elongation, or termination factors, as well as ARSases) and ribosomal proteins across tissues, with a few notable exceptions, such as the ovary (where mRNAs encoding ribosomal proteins turned out to be over-represented likely due to accelerated ribosome production during oocytes maturation).

Our approach allowed comparing the relative concentration of translation factors and revealed some tissue-specific peculiarities in the composition of protein synthesis machinery that can contribute to translational landscapes of human tissues. For example, we would like to note a potentially higher total activity of eIF1, eIF1A, and BZW in the heart due to an “additional source” of these factors, i.e., the elevated expression of *EIF1B*, *EIF1AX*, and *BZW2* genes specifically in this organ. These factors are known to enhance the stringency of start site selection [45,91] and thus should remodel a pattern of efficiently translated mRNAs. Future studies using ribosome profiling of animal organs could shed some light on this issue [2,12,13,14,15].

We would also like to note that some translation machinery components may have moonlighting functions, being involved in unrelated processes [109]. Thus, some of the observed differences may reflect these additional activities rather than tissue-specific features of protein synthesis. The cases presented in this study can provide a starting point for further research in this intriguing direction.

Our study also draws attention to sexual dimorphism in the abundance of some translation machinery components. At least three important factors, eIF1A, eIF2γ, and DDX3, are encoded in sex chromosomes (by the X-linked *EIF1AX*, *DDX3X*, and *EIF2S3* genes, and Y-linked *EIF1AY* and *DDX3Y* genes). In addition to the predictable lack of the Y-linked gene expression in female organs, this issue clearly contributes to total amounts of translation factors in some organs (e.g., the higher eIF1A level in the heart due to a “double portion” of EIF1AX/Y expression, or the lower eIF2γ level in the testis than in female organs due to *EIF2S3* X-inactivation escape).

Our analysis identified the testis and brain as organs with the most diverged expression of TAGs. Although it is well known that neurons and nervous tissues have peculiarities in protein synthesis [110,111], translation regulation in the testis has not yet been extensively investigated. A recent study [2] found that the correlation between transcriptome and translatome in the testis is much lower than in the brain and liver and revealed a unique pattern in the testis that is explained by strong differential regulation of translation across spermatogenic cell types. The authors also found that translational upregulation specifically counterbalanced the effects of meiotic sex-chromosome inactivation during spermatogenesis [2]. It should also be noted that the brain and testis are immunologically privileged organs, and this may bring some bias in transcriptomic and proteomic analysis of their content. It was also shown recently that the testis and brain express genes that are enriched in rare codons both in humans and flies [112].

Recent studies show that different tissues can have different tRNA repertoires and codon usage [16,17]. The adaptation of the tRNA pool was found to be largely related to a tissue proliferative state [16]. Then, two clusters of tissues with an opposite pattern of codon preferences were identified [113]: some tissues (including kidney, muscle, heart, liver, colon, fat, and ovary) generally favor C/G-ending codons, while others (including lung, brain, pancreas, spleen, small intestine, adrenal and salivary glands, placenta, and testis) better tolerate translation of rare A/T-ending codons. Although we were unable to find any obvious correlation between these clusters and the distribution of elongation factors or ARSases, a more in-depth analysis may reveal such patterns in future.

Finally, we would like to emphasize the importance of using both transcriptomic and proteomic analyses. Although mRNA levels are primary determinants for protein abundance (for review, see [114]), translation regulation can significantly contribute to the levels of translation machinery components [93]. Unfortunately, for many poorly expressed genes, proteomic data are either lacking or statistically unreliable, while transcriptomic datasets from GTEx, FANTOM, and Gene Atlas are usually enriched in reliable information about most human genes. The discrepancy between the transcript and protein levels that we reported for some translation-associated genes can be a starting point for the investigation of their post-transcription regulation.

## 4. Materials and Methods

Data analysis and visualization were conducted in R environment (R 4.2.2). We analyzed mRNA abundance data of different tissues and primary cells available in FANTOM5 (http://fantom.gsc.riken.jp/5/, accessed on 5 March 2023), GTEx (https://gtexportal.org/, accessed on 5 March 2023), and Gene Atlas [101] databases, while mass-spectrometry data were taken from [1]. We considered the genes encoding protein synthesis machinery components according to Table 1.

The CAGE data (cap analysis of gene expression) from FANTOM5 (http://fantom.gsc.riken.jp/5/) phase 1 mapped to hg19 genome assembly were downloaded as normalized TPM (Tags Per Million) for each transcript, as provided in the FANTOM5 data, and summed up across transcripts to obtain the gene-level expression estimates. Samples with extreme normalization factors (less than 0.7 or higher than 1.4) were excluded from the analysis. The Gene Atlas data [101] were obtained from the BioGPS portal (http://biogps.org/, accessed on 5 March 2023) as normalized expression units from the Human U133A/GNF1H microarray. Probe sets represented in U133A annotation were selected for further analysis, and the mean expression across probes was considered as the estimate of gene expression. GTEx v7 RNASeQCv1.1.8 gene expression data were downloaded as TPM (Transcripts Per Kilobase Million) from https://gtexportal.org/home/datasets, accessed on 5 March 2023.

In our analysis, we used absolute gene expression estimates as well as relative expression estimated as a share of a particular gene in the total gene expression of a particular complex (see Table 1). Relative estimates were used to analyze the target gene expression in the context of a protein complex with a particular functional role. For the latter analysis, we classified the gene complexes according to the functional and physical interactions of the respective proteins (Table 1). The use of relative gene expression allowed us to address the changes in relative transcript abundance in the context of the expression of protein partners and the stoichiometry of the complexes.

To obtain a reliable statistical estimate of the tissue-specific contribution of a particular protein to a particular complex or group, we used GTEx data and the following approach. First, for each complex, we excluded the samples with the total expression of genes of the complex less than half a mean across samples. Next, for each member of the complex, we estimated its share in the total expression of the complex. With the vector of shares across samples, we performed set enrichment analysis on the ranked list of samples. To this end, we classified the samples according to the tissue of origin, e.g., ‘brain’ (30 groups of 11,688 samples in total according to GTEx metadata), and checked the positions of samples of a particular group in the total ranked list of samples. Normalized enrichment scores (NES) and statistical significance were estimated with fgsea R package [115] (10,000 permutations). *p*-values were corrected for multiple testing using FDR correction for the number of sample groups.

To calculate correlation of the mean expression of genes encoding ribosomal proteins (Supplementary Table S1) and the mean expression of the major translation-associated gene sets in various human tissues, the weighted total expression for each tissue was calculated as a sum of weighted expression values for each gene estimated as gene expression values (TPM) normalized to the mean of expression values for the gene in every sample. Pearson correlation was computed with R.

## 5. Conclusions

In summary, in this study, we systematically explored the tissue-specific expression of translation-associated genes and found new cases of differentially represented translation factors and auxiliary proteins in human organs and tissues. These findings contribute to our understanding of translational control in health and disease.

## Figures and Tables

**Table 1:** Complexes and functional groups of translation-associated proteins classified according to functional and physical interactions.

Complex or Group	Protein Names
eIF2	EIF2S1, EIF2S2, EIF2S3
eIF2B	EIF2B1, EIF2B2, EIF2B3, EIF2B4, EIF2B5
eIF3	EIF3A, EIF3B, EIF3C, EIF3D, EIF3E, EIF3F, EIF3G, EIF3H, EIF3I, EIF3J, EIF3J, EIF3K, EIF3L, EIF3M
Multifactor complex (MFC)	EIF1, EIF1AX, EIF1AY, EIF2B1, EIF2B2, EIF2B3, EIF2B4, EIF2B5, EIF2S1, EIF2S2, EIF2S3, EIF3A, EIF3B, EIF3C, EIF3D, EIF3E, EIF3F, EIF3G, EIF3H, EIF3I, EIF3J, EIF3J, EIF3K, EIF3L, EIF3M, EIF5
eIF4s	EIF4A1, EIF4A2, EIF4B, EIF4H, EIF4E, EIF4E1B, EIF4E3, EIF4G1, EIF4G3
eIF4, 4EBP	EIF4EBP1, EIF4EBP2, EIF4EBP3, EIF4A1, EIF4A2, EIF4B, EIF4H, EIF4E, EIF4E1B, EIF4E3, EIF4G1, EIF4G3
INITIATION	EIF1, EIF1AX, EIF1AY, EIF1B, EIF2B1, EIF2B2, EIF2B3, EIF2B4, EIF2B5, EIF2S1, EIF2S2, EIF2S3, EIF3A, EIF3B, EIF3C, EIF3D, EIF3E, EIF3F, EIF3G, EIF3H, EIF3I, EIF3J, EIF3J, EIF3K, EIF3L, EIF3M, EIF4A1, EIF4A2, EIF4B, EIF4H, EIF4E, EIF4E1B, EIF4E3, EIF4G1, EIF4G3, DDX3X, DDX3Y, DHX29, EIF4EBP1, EIF4EBP2, EIF4EBP3, EIF5, EIF5B, PABPC1
INITIATION+	EIF1, EIF1AX, EIF1AY, EIF1B, EIF2B1, EIF2B2, EIF2B3, EIF2B4, EIF2B5, EIF2S1, EIF2S2, EIF2S3, EIF3A, EIF3B, EIF3C, EIF3D, EIF3E, EIF3F, EIF3G, EIF3H, EIF3I, EIF3J, EIF3J, EIF3K, EIF3L, EIF3M, EIF4A1, EIF4A2, EIF4B, EIF4H, EIF4E, EIF4E1B, EIF4E3, EIF4G1, EIF4G3, DDX3X, DDX3Y, DHX29, EIF4EBP1, EIF4EBP2, EIF4EBP3, EIF5, EIF5B, EIF6, PABPC1, ABCE1, EIF2D, MCTS1, DENR, MCTS2P, BZW1, BZW2, EIF4E2, EIF4G2, CTIF, PAIP1, PAIP2, EIF2A, ABCF1, NCBP1, NCBP2
eEF1B	EEF1B2, EEF1D, EEF1G
eEF1H	EEF1A1, EEF1A2, EEF1B2, EEF1D, EEF1G, VARS1
ELONGATION	EEF1A1, EEF1A2, EEF1B2, EEF1B2P2, EEF1D, EEF1G, EEF2, EEFSEC, EIF5A, EIF5A2
ELONGATION+	EEF1A1, EEF1A2, EEF1B2, EEF1B2P2, EEF1D, EEF1G, EEF2, DPH1, DPH2, DPH3, DPH5, DPH6, DPH7, EEFSEC, EIF5A, EIF5A2, EIF5AL1, DHPS, DOHH
ELONGATION+ GTPBPs	GTPBP1, GTPBP2, EEF1A1, EEF1A2, EEF1B2, EEF1B2P2, EEF1D, EEF1G, EEF2, DPH1, DPH2, DPH3, DPH5, DPH6, DPH7, EEFSEC, EIF5A, EIF5A2, EIF5AL1, DHPS, DOHH
TERMINATION	ETF1, GSPT1, GSPT2
TERMINATION+	ETF1, GSPT1, GSPT2, PABPC1, ABCE1, EIF2D, MCTS1, DENR, MCTS2P, HBS1L, PELO
PABPC paralogs	PABPC1L, PABPC1L2A, PABPC1L2B, PABPC3, PABPC4, PABPC4L, PABPC5, PABPC1
ARSases	AIMP1, AIMP2, EEF1E1, NARS, RARS, EPRS, MARS, QARS, IARS, KARS, LARS, YARS, VARS, AARS, CARS, DARS, FARSA, FARSB, GARS, HARS, HARS2, SARS, SARS2, TARS, TARSL2, WARS, LARS2, RARS2, VARS2, AARS2, TARS2, YARS2, WARS2, NARS2, PARS2, MARS2, IARS2, FARS2, EARS2, DARS2, CARS2
ARSase COMPLEX	AIMP1, AIMP2, EEF1E1, NARS, RARS, EPRS, MARS, QARS, IARS, KARS, LARS

## Data Availability

Not applicable.

## Supplementary Materials

The following supporting information can be downloaded at: https://www.mdpi.com/article/10.3390/ijms24098361/s1.
