# Supplementary material for: Human Tissues Exhibit Diverse Composition of Translation Machinery
Source: Int J Mol Sci. 2023 May 6;24(9):8361. doi: 10.3390/ijms24098361 (PMC10179197; doi:10.3390/ijms24098361)
Supplement: Supplementary file 1 [file ijms-24-08361-s001.zip › ijms-2293172-supplementary.pdf]

Human tissues exhibit diverse composition of translation machinery

Aleksandra S. Anisimova, Natalia M. Kolyupanova, Nadezhda E. Makarova, Artyom A. Egorov, Ivan V. Kulakovskiy, Sergey E. Dmitriev

SUPPLEMENTARY TABLES AND FIGURES

Supplementary Table S1. Human genes encoding ribosomal proteins used in the study.

Group	Gene names
RPSs	*RPSA, RPS2, RPS3, RPS3A, RPS4X, RPS4Y1, RPS4Y2, RPS5, RPS6, RPS7, RPS8, RPS9, RPS10, RPS11, RPS12, RPS13, RPS14, RPS15, RPS15A, RPS16, RPS17, RPS18, RPS19, RPS20, RPS21, RPS23, RPS24, RPS25, RPS26, RPS27, RPS27A, RPS27L, RPS28, RPS29, FAU*
RPLs	*RPLP0, RPLP1, RPLP2, RPL3, RPL3L, RPL4, RPL5, RPL6, RPL7, RPL7A, RPL7L1, RPL8, RPL9, RPL10, RPL10A, RPL10L, RPL11, RPL12, RPL13, RPL13A, RPL14, RPL15, RPL17, RPL18, RPL18A, RPL19, RPL21, RPL22, RPL22L1, RPL23, RPL23A, RPL24, RPL26, RPL26L1, RPL27, RPL27A, RPL28, RPL29, RPL30, RPL31, RPL32, RPL34, RPL35, RPL35A, RPL36, RPL36A, RPL36AL, RPL37, RPL37A, RPL38, RPL39, RPL39L, UBA52, RPL41*

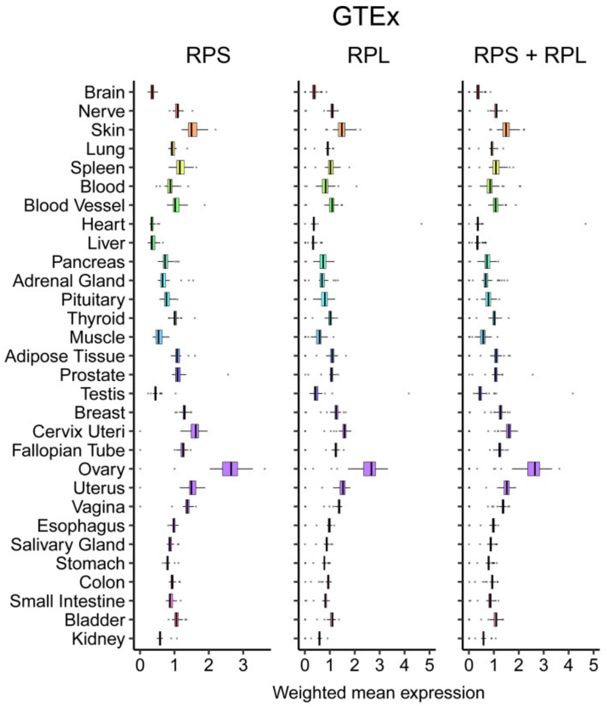

Supplementary Figure S1. Expression of the human genes encoding ribosomal proteins (RPS, proteins of the small subunit; RPL, proteins of the large subunit; RPS+RPL, both) across tissues and organs. Boxplots show the geometric mean of transcript abundance (transcripts per million, TPM) in GTEx samples corresponding to the indicated tissues and organs.

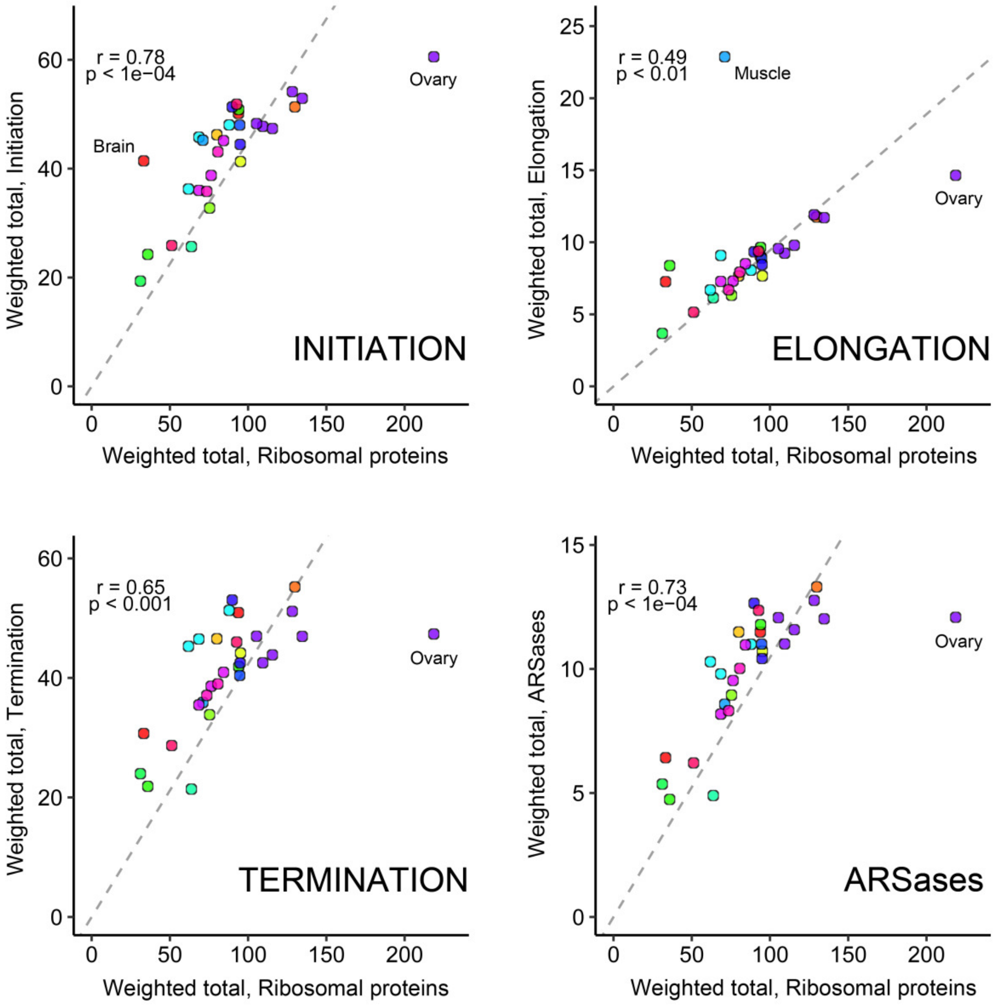

Supplementary Figure S2. Correlation of the mean expression of genes encoding ribosomal proteins and the mean expression of the major translation-associated gene sets in various human tissues. The weighted total expression for each tissue was calculated as a sum of weighted expression values for each gene estimated as gene expression values (TPM) normalized to the mean of expression values for the gene in every sample. Pearson correlation was computed with R and indicated on the plots; linear regression slope is shown as the dashed line. Selected outliers are explicitly labeled.

Tissue-specific composition of translation machinery

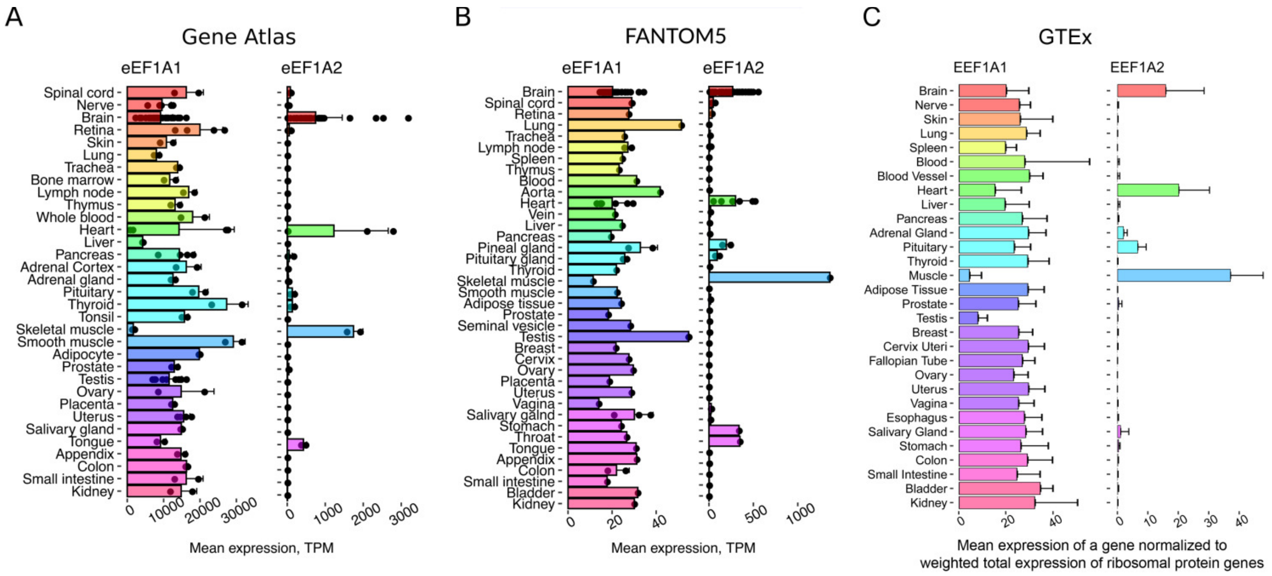

Supplementary Figure S3. Tissue-specific expression pattern of two human genes encoding eEF1A paralogs, *EEF1A1* and *EEF1A2*. **(A)** Expression of the *EEF1A1* and *EEF1A2* genes in various human tissues according to Gene Atlas [1]. **(B)** Expression of the *EEF1A1* and *EEF1A2* genes in various human tissues according to FANTOM5. **(C)** Relative expression of *EEF1A1* and *EEF1A2* estimated with an alternative normalization: weighted total expression of ribosomal protein genes was used to normalize the abundance of the *EEF1A1* and *EEF1A2* transcripts, instead of the total expression of the “ELONGATION” complex genes used in Figure 1D. TPM, Transcripts Per Kilobase Million.

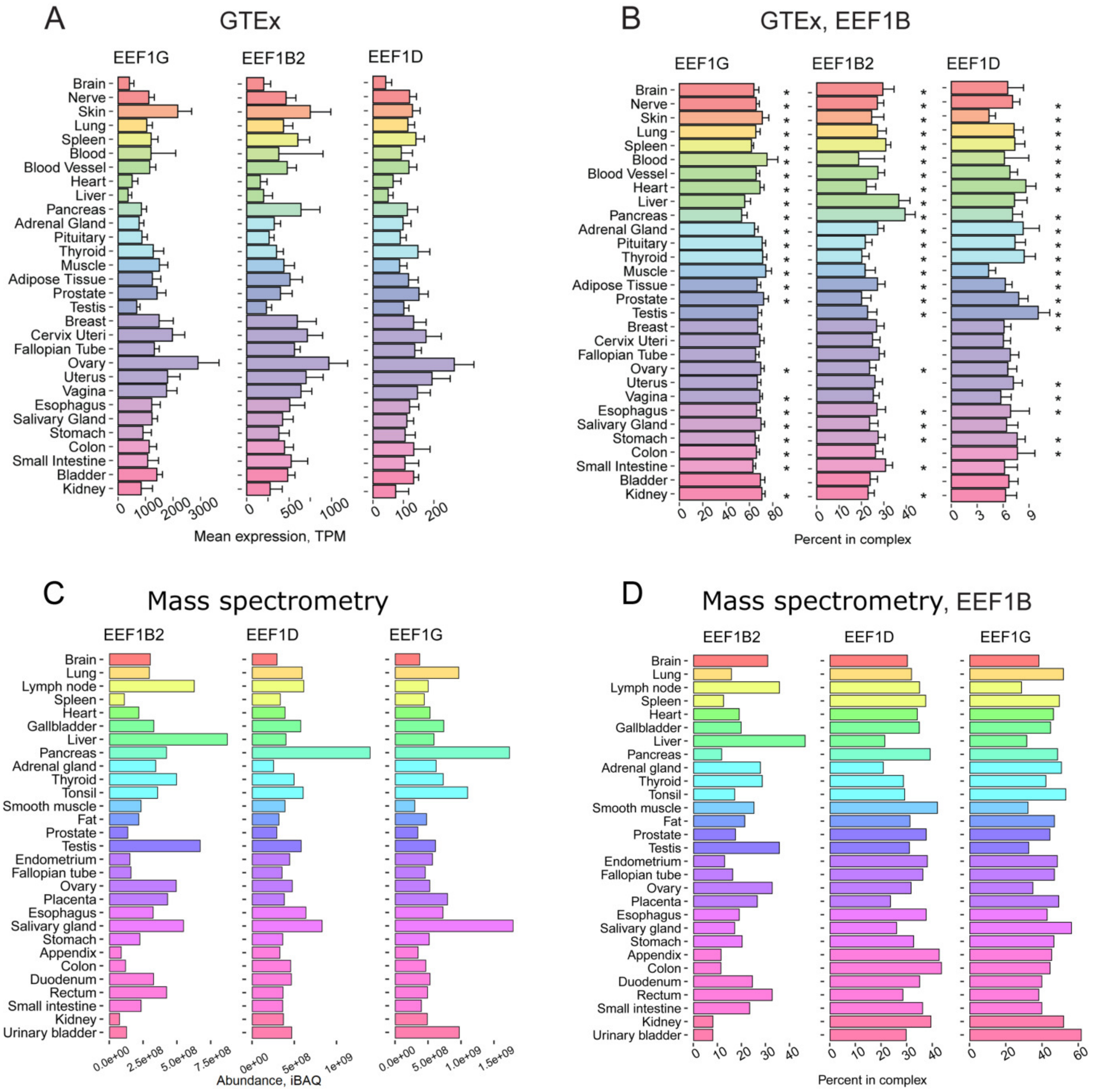

Supplementary Figure S4. Expression pattern of the human genes encoding eEF1B subunits. **(A)** Expression of the *EEF1B2*, *EEF1D*, and *EEF1G* genes (encoding eEF1Bα, eEF1Bδ, and eEF1Bγ, respectively) in various human tissues according to GTEx. TPM, Transcripts Per Kilobase Million. **(B)** Relative abundances of the *EEF1B2*, *EEF1D*, and *EEF1G* transcripts (normalized to their sum). **(C)** Levels of the eEF1Bα, eEF1Bδ, and eEF1Bγ proteins in various human tissues according to high-throughput proteomic analysis [2]. **(D)** The abundances of eEF1B subunits relative to their sum.

Tissue-specific composition of translation machinery

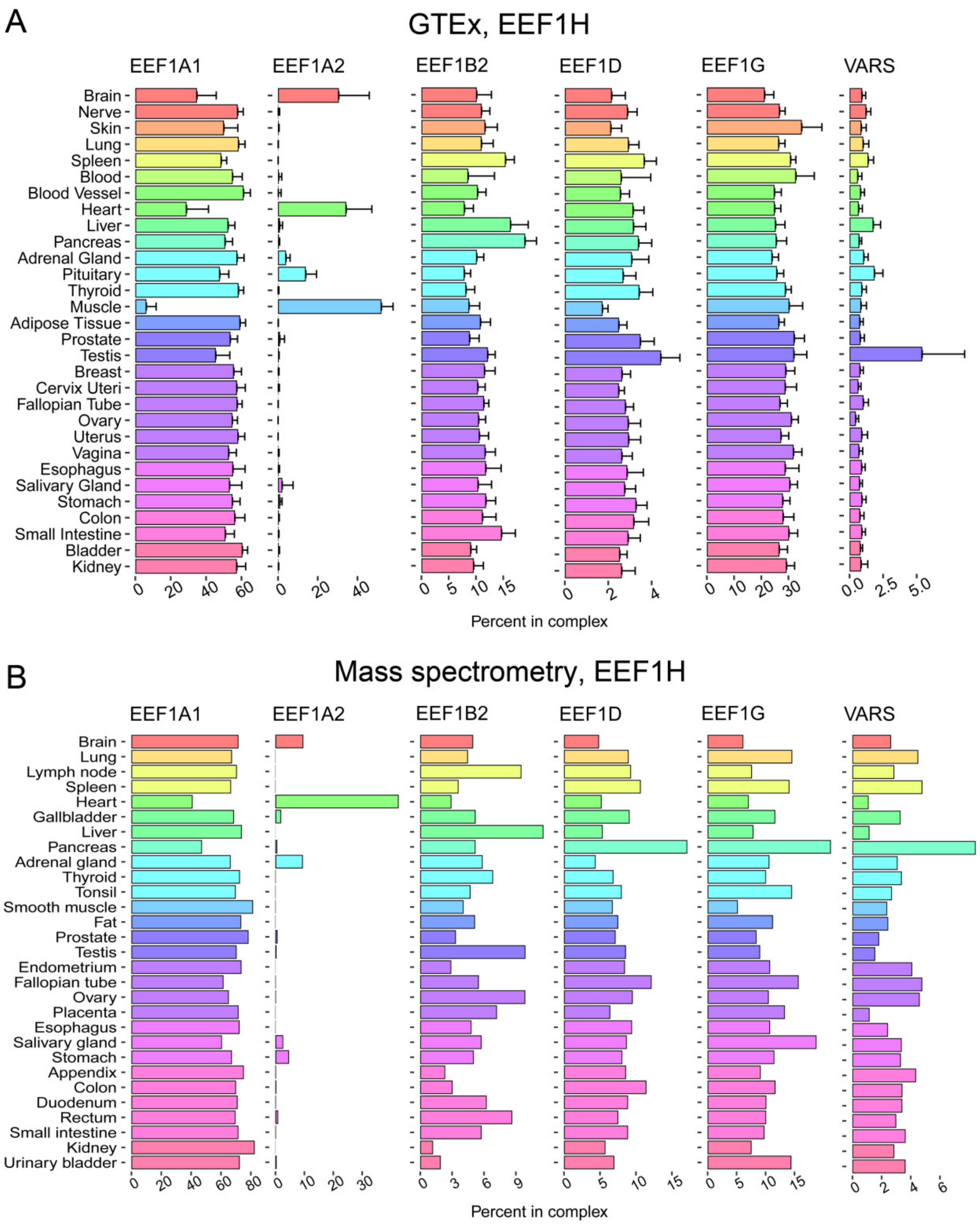

Supplementary Figure S5. Relative expression of genes encoding the eEF1H components at the mRNA **(A)** and protein levels **(B)** according to GTEx transcriptome database or the high-throughput proteomic analysis [2], respectively.

Tissue-specific composition of translation machinery

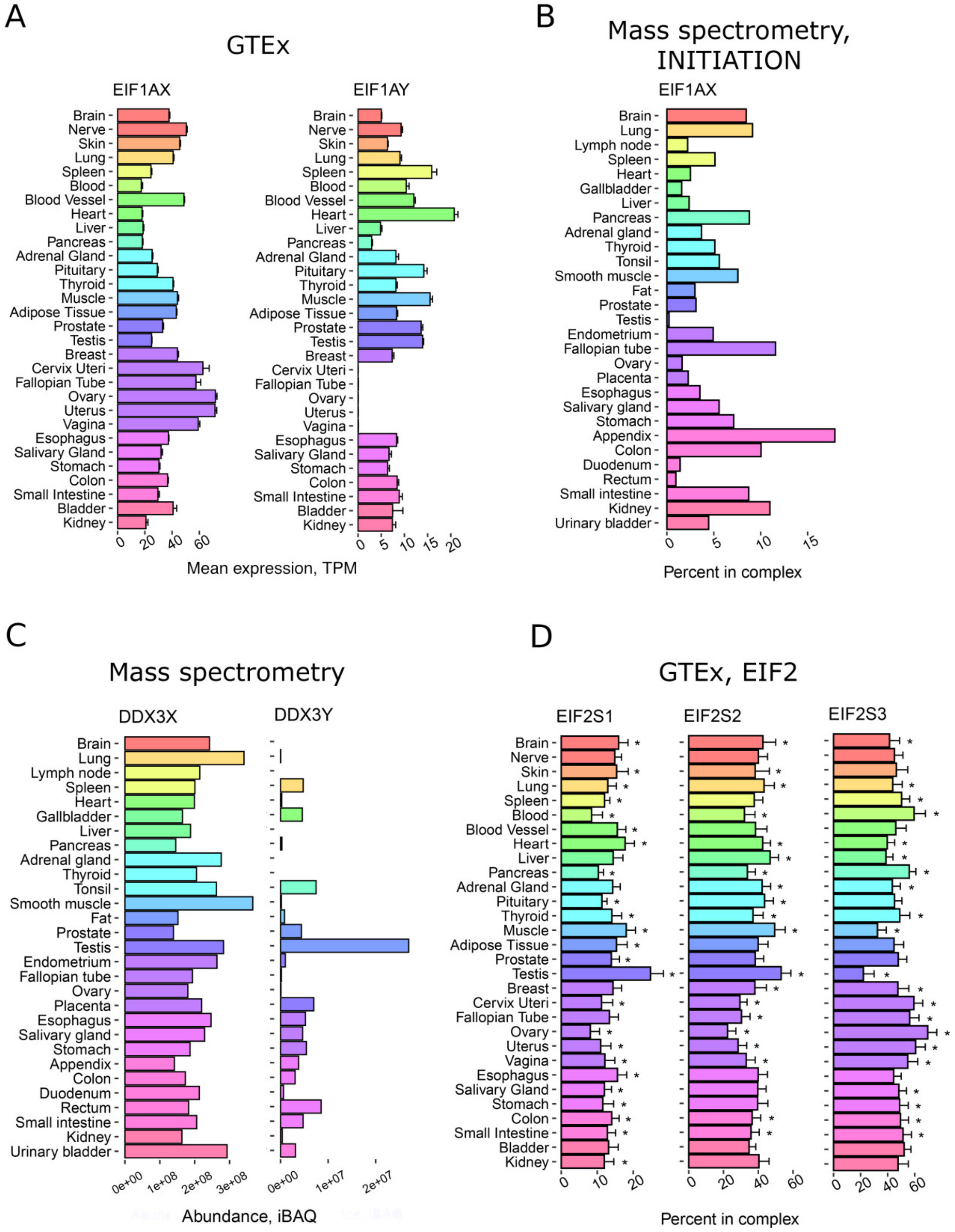

Supplementary Figure S6. Tissue-specific expression pattern of translation-associated genes localized in sex chromosomes. **(A)** Expression of the *EIF1AX* and *EIF1AY* genes in various human tissues according to GTEx. **(B)** Percentage of *EIF1AX* expression among the genes from the "INITIATION" complex across various human tissues, according to proteomic analysis [2]. **(C)** Levels of DDX3X and DDX3Y proteins in various human tissues according to proteomic analysis. **(D)** Percentage of *EIF2S1*, *EIF2S2*, and *EIF2S3* expression encoding α, β, and γ-subunits of eIF2 within the "eIF2" complex across various human tissues, according to GTEx. *, FDR corrected p-value < 0.01 in enrichment analysis (fgsea R package).

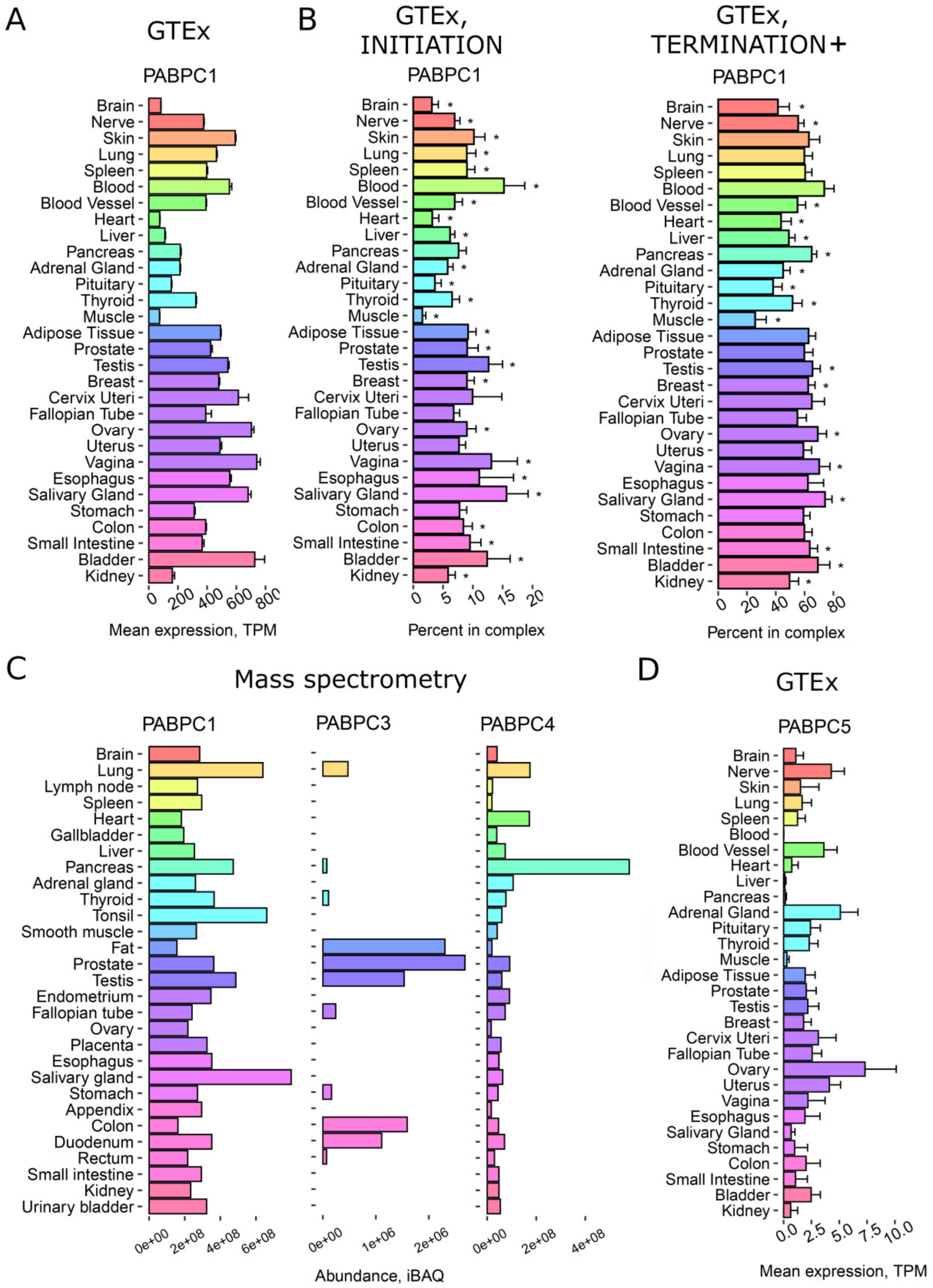

Supplementary Figure S7. Tissue-specific expression pattern of genes encoding PABPC homologs. **(A)** Expression of the *PABPC1* in various human tissues according to GTEx. **(B)** Percentage of *PABPC1* expression among the genes from the "INITIATION" and "TERMINATION +" complexes across various human tissues, according to GTEx. **(C)** Levels of PABPC1, PABPC3, and PABPC4 proteins in various human tissues according to proteomic analysis [2]. **(D)** Expression of the *PABPC5* in various human tissues according to GTEx. TPM, Transcripts Per Kilobase Million; *, FDR corrected p-value < 0.01 in enrichment analysis (fgsea R package).

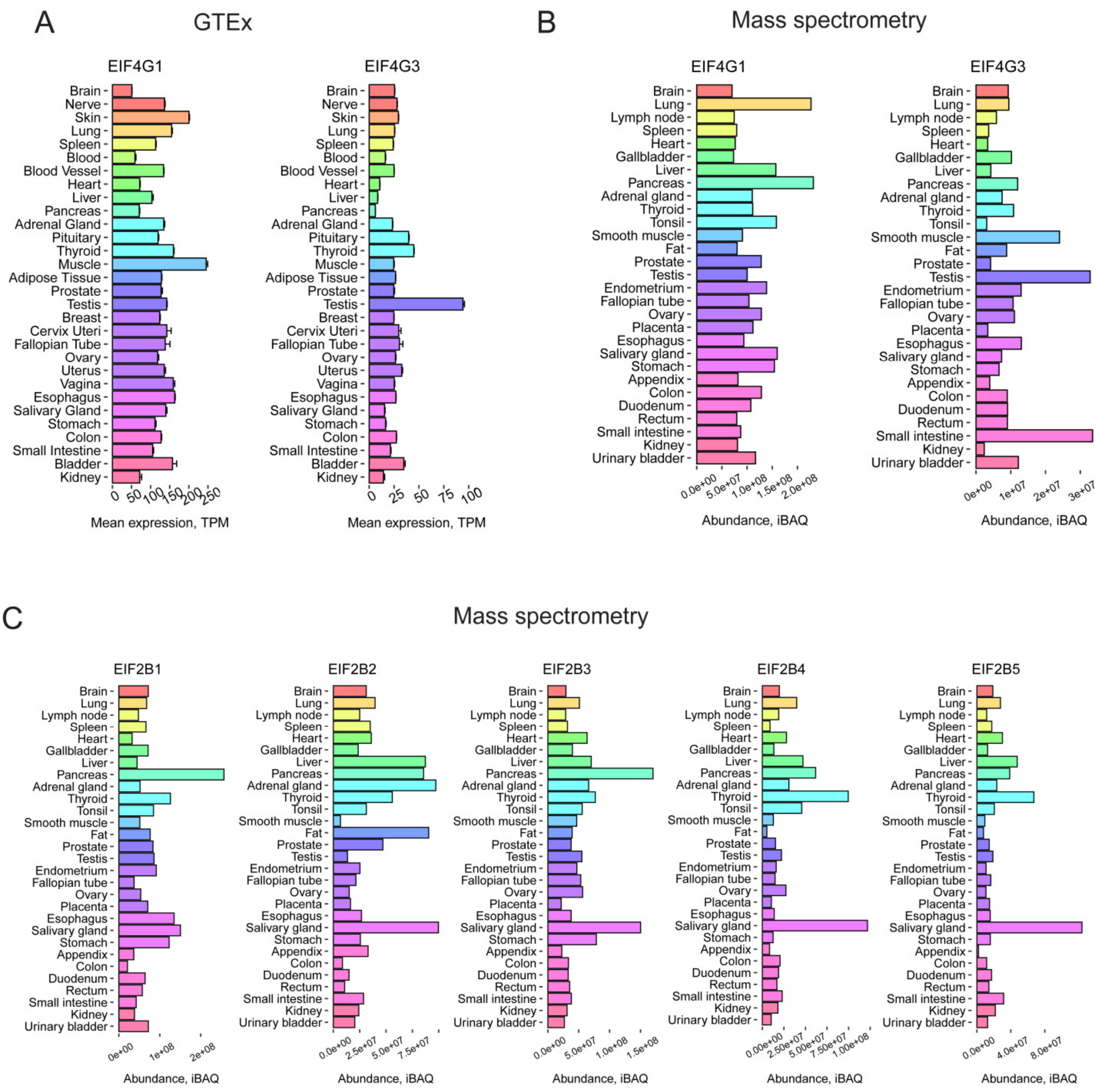

Supplementary Figure S8. Tissue-specific expression pattern of genes encoding GTPBP1 and GTPBP2 proteins, eIF4G paralogs, and eIF2B subunits. **(A)** Expression of the *EIF4G1* and *EIF4G3* genes in various human tissues according to GTEx. **(B)** Levels of EIF4G1 and EIF4G3 proteins in various human tissues according to proteomic analysis [2]. **(C)** Levels of eIF2B subunits in various human tissues according to proteomic analysis. TPM, Transcripts Per Kilobase Million.

Tissue-specific composition of translation machinery

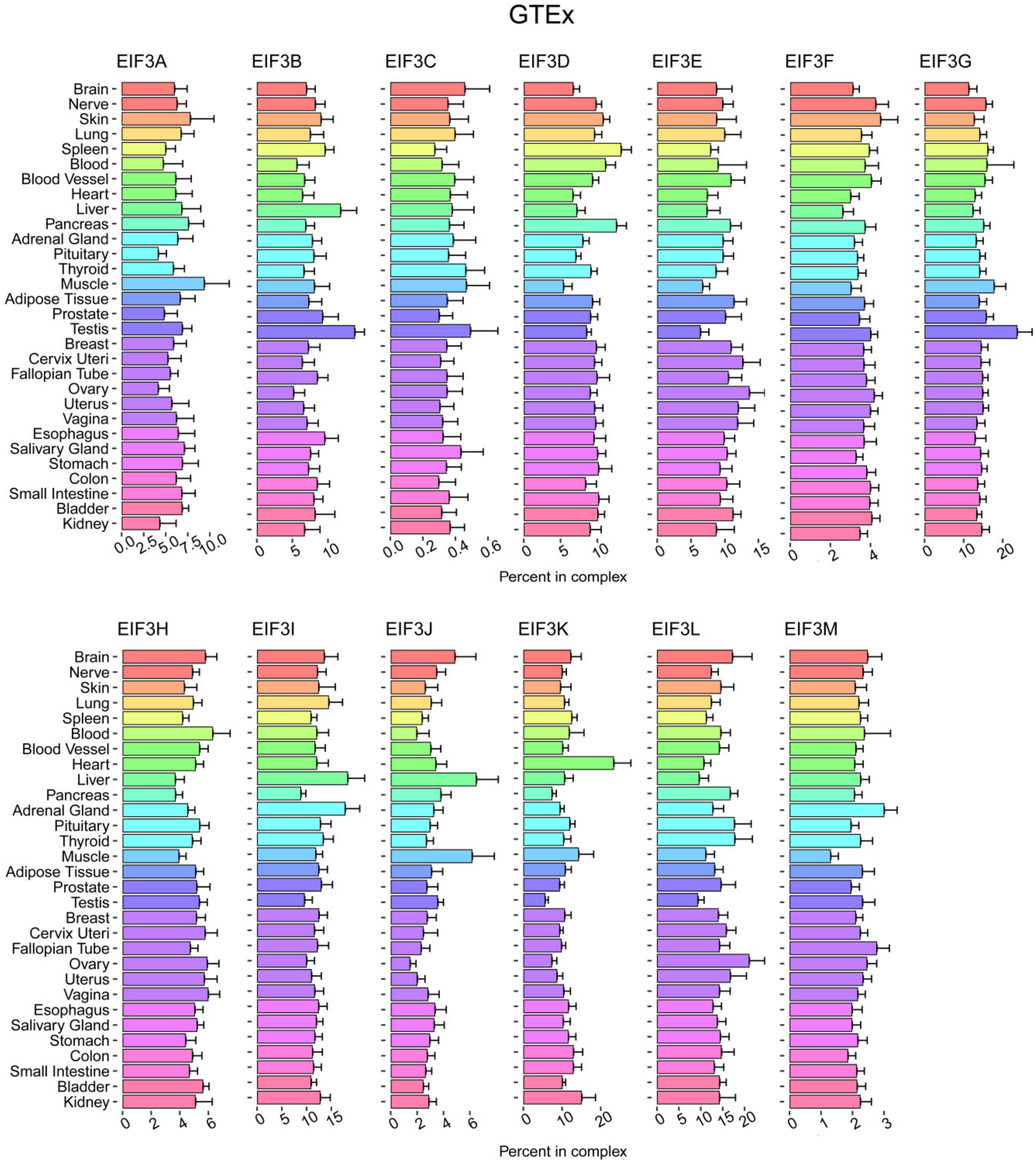

Supplementary Figure S9. Tissue-specific expression pattern of genes encoding human eIF3. Percentage of gene expression within the "eIF3" complex across various human tissues, according to GTEx.

Tissue-specific composition of translation machinery

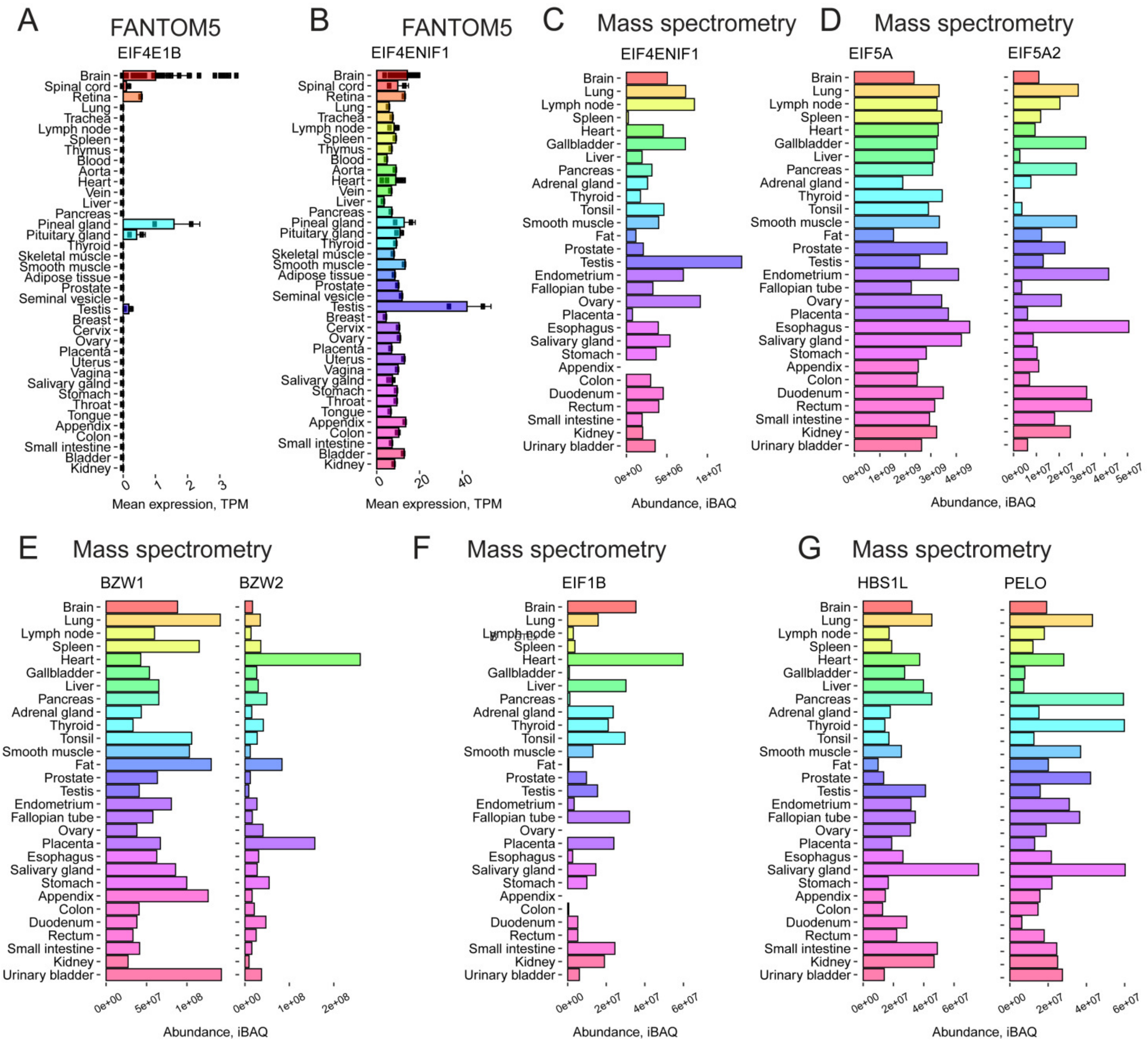

Supplementary Figure S10. Tissue-specific expression pattern of translation-associated genes showing high tissue specificity. **(A)** Expression of the *EIF4E1B* in various human tissues according to FANTOM5. **(B)** Expression of the *EIF4ENIF1* in various human tissues according to FANTOM5. **(C)** Levels of EIF4ENIF1 protein in various human tissues according to proteomic analysis [2]. **(D)** Levels of eIF5A homologs in various human tissues according to proteomic analysis. **(E)** Levels of BZW1/5MP2 and BZW2/5MP1 proteins in various human tissues according to proteomic analysis.**(F)** Levels of eIF1B protein in various human tissues according to proteomic analysis. **(G)** Levels of HBS1L and PELO proteins in various human tissues according to proteomic analysis. TPM, Transcripts Per Kilobase Million.

Tissue-specific composition of translation machinery

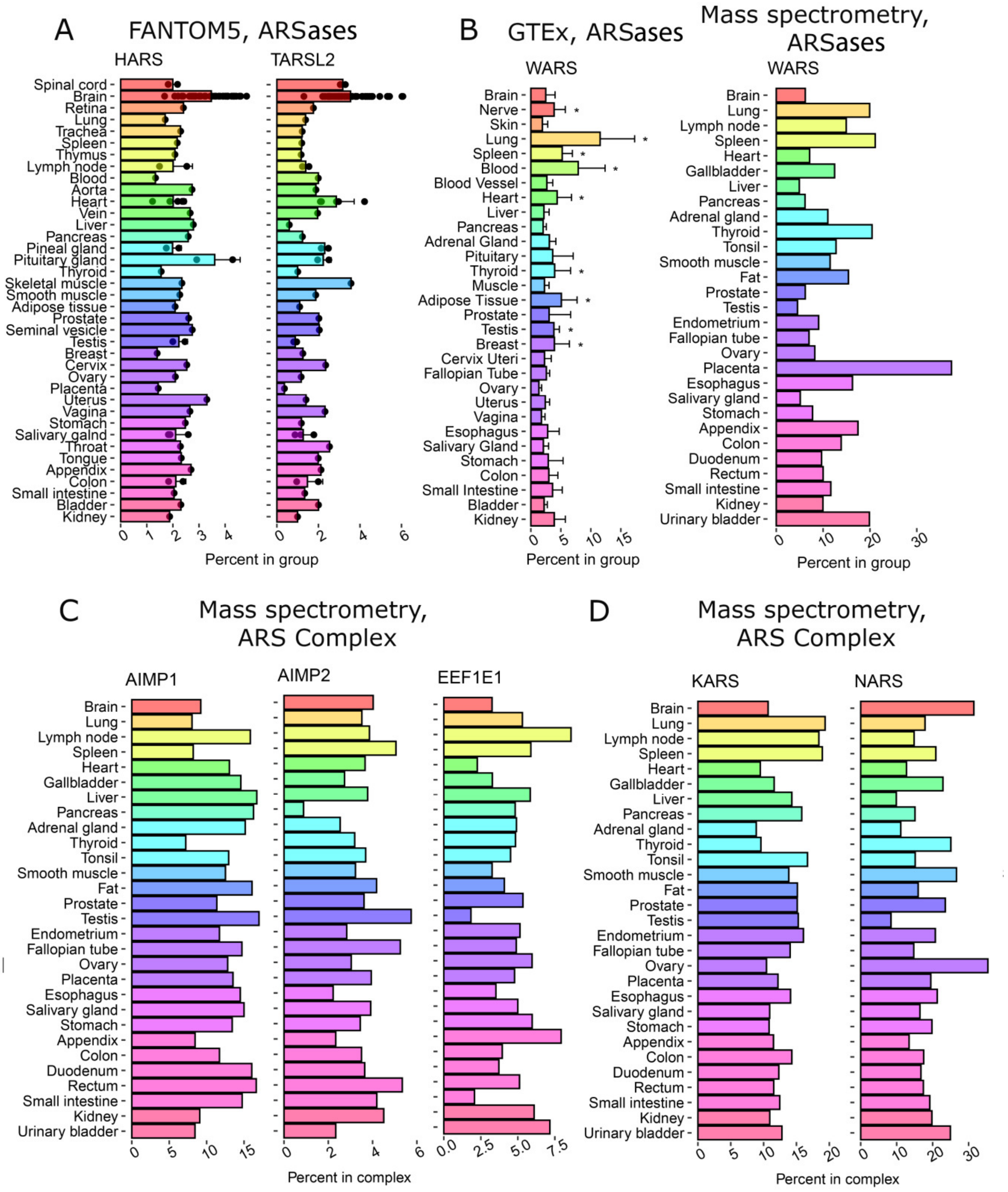

Supplementary Figure S11. Expression pattern of several genes encoding aminoacyl-tRNA-synthetases (ARSases) with pronounced tissue specificity. **(A)** Percentage of *HARS* and *TARSL2* expression among the genes from the "ARSases" complex across various human tissues, according to FANTOM5. **(B)** Percentage of *WARS* expression among the genes from the "ARSases" complex across various human tissues, according to GTEx and proteomic analysis [2]. **(C)** Percentage of *AIMP1*, *AIMP2*, and *EEF1E1* expression among the genes from the "ARSase COMPLEX" complex across various human tissues, according to proteomic analysis. **(D)** Percentage of *KARS* and *NARS* expression among the genes from the "ARSase COMPLEX" complex across various human tissues, according to proteomic analysis. TPM, Transcripts Per Kilobase Million; *, FDR corrected p-value < 0.01 in enrichment analysis (fgsea R package).

References

1. Su, A.I.; Wiltshire, T.; Batalov, S.; Lapp, H.; Ching, K.A.; Block, D.; Zhang, J.; Soden, R.; Hayakawa, M.; Kreiman, G.; Cooke, M.P.; Walker, J.R.; Hogenesch, J.B. A gene atlas of the mouse and human protein-encoding transcriptomes. *Proc Natl Acad Sci U S A.* **2004,** *101*, 6062-7.

2. Wang, D.; Eraslan, B.; Wieland, T.; Hallstrom, B.; Hopf, T.; Zolg, D.P.; Zecha, J.; Asplund, A.; Li, L.H.; Meng, C.; Frejno, M.; Schmidt, T.; Schnatbaum, K.; Wilhelm, M.; Ponten, F.; Uhlen, M.; Gagneur, J.; Hahne, H.; Kuster, B. A deep proteome and transcriptome abundance atlas of 29 healthy human tissues. *Mol Syst Biol.* **2019,** *15*, e8503.
